# Candidate gene study to investigate the genetic determinants of normal variation in central corneal thickness

**Published:** 2010-03-31

**Authors:** David P. Dimasi, Kathryn P. Burdon, Alex W. Hewitt, Ravi Savarirayan, Paul R. Healey, Paul Mitchell, David A. Mackey, Jamie E. Craig

**Affiliations:** 1 Department of Ophthalmology, Flinders University, Adelaide, South Australia, Australia; 2 Centre for Eye Research Australia, University of Melbourne, Royal Victorian Eye and Ear Hospital, Melbourne, Victoria, Australia; 3 Murdoch Childrens Research Institute, Genetic Health Services Victoria, and Department of Paediatrics, University of Melbourne, Parkville, Victoria, Australia; 4 Centre for Vision Research, Department of Ophthalmology and Westmead Millennium Institute, University of Sydney, Westmead, New South Wales, Australia; 5 Centre for Ophthalmology and Visual Science, University of Western Australia, Perth, Western Australia, Australia

## Abstract

**Purpose:**

The genetic component underlying variation in central corneal thickness (CCT) in the normal population remains largely unknown. As CCT is an identified risk factor for open-angle glaucoma, understanding the genes involved in CCT determination could improve our understanding of the mechanisms involved in this association.

**Methods:**

To identify novel CCT genes, we selected eight different candidates based on a range of criteria. These included; aquaporin 1 (*AQ1*), aquaporin 5 (*AQ5*), decorin (*DCN*), fibrillin-1 (*FBN1*), keratocan (*KERA*), lumican (*LUM*), osteoglycin (*OGN*), and paired box 6 (*PAX6*). Tagging single nucleotide polymorphisms (SNPs) selected from the HapMap database were genotyped to cover the majority of genetic variation within each gene. Each SNP was screened in a large, population-based cohort from Australia and both single SNP and haplotype analyses were undertaken.

**Results:**

Two SNPs were found to be nominally associated with CCT, rs17352842 from *FBN1* (p=0.02) and rs3026398 from *PAX6* (p=0.02), although neither of these p values survived correction for multiple testing. Haplotype analysis revealed one haplotype within *FBN1* (corrected p=0.048) and two haplotypes within *PAX6* (strongest corrected p=0.006) associated with CCT. No other SNPs or haplotypes from the remaining genes showed any significant correlation with CCT.

**Conclusions:**

Results from this study suggest that *FBN1* and *PAX6* are potentially involved in determining CCT. This is the first published study to investigate these genes for association with normal CCT variation.

## Introduction

The identification of central corneal thickness (CCT) as a risk factor for the potentially blinding condition open-angle glaucoma (OAG) has stimulated interest in understanding the genetic mechanisms of this trait. Evidence from numerous studies has identified lower CCT measurements as a risk factor for progression from ocular hypertension to OAG [1-3], greater severity of visual field damage [4,5], and more rapid progression of established visual field loss [6-8]. Interestingly, several of these studies also demonstrate that this relationship is independent of the known confounding effect CCT has on intra-ocular pressure measurements [1,3,8], suggesting that the association between CCT and OAG may be more than just tonometry artifact. If a genuine biologic interaction exists between structures in the cornea and those important in OAG pathology, then this raises the possibility that genes involved in the determination of CCT may also predispose to OAG.

Within the general population, CCT is a normally distributed quantitative trait, with evidence from twin and familial studies indicating that it is highly heritable [9-11]. However, despite its high heritability, little is known about the genes that contribute to normal CCT variation within the general population. Recently, a candidate gene study identified an association with the type I collagen genes *COL1A1* and *COL1A2* and normal CCT variation, but these have been the only genes identified to-date [12]. These genes were selected based on the observation that patients with the connective tissue disorder osteogenesis imperfecta, which is caused by mutations in *COL1A1* and *COL1A2*, have abnormally thin CCT measurements [13,14]. There are several other genetic conditions that exhibit abnormal CCT measurements, including Marfan syndrome [15,16] and aniridia [17,18], thus implicating the defective genes, fibrillin-1 (*FBN1*) [19,20] and paired box 6 (*PAX6*) [21,22], respectively, as potential modifiers of CCT.

Apart from identifying genes for normal CCT variation based on observations in genetic disorders, structural and metabolic proteins within the cornea also offer plausible candidates. The predominant layer of the cornea, the corneal stroma, is largely comprised of extracellular material including collagens and proteoglycans. A family of proteins termed the small leucine-rich proteoglycans (SLRPs) are prominent within the stroma and have a pivotal role in maintaining the structural integrity of the extracellular matrix, which is vital for corneal transparency. Some of these SLRPs include decorin (DCN), keratocan (KERA), lumican (LUM), and osteoglycin (OGN). Evidence from human and animal studies also suggests these proteins may play a role in the determination of CCT. Mutations in *DCN* result in a disorder known as congenital corneal stromal dystrophy, which causes an abnormally thick cornea [23]. Mice with *KERA* and *LUM* gene knockouts have thin corneal stromas [24-26], while *OGN* knockout mice have thicker corneal collagen fibrils [27]. Another group of proteins that may play a role in CCT determination are the aquaporins. Mice carrying an aquaporin 1 (*AQP1*) gene knockout exhibit a decreased CCT, while mice with an aquaporin 5 (*AQP5*) knockout show an increased CCT [28].

In this study, we aimed to evaluate each of these candidate genes (*AQP1, AQP5, DCN, FBN1, KERA, LUM, OGN*, and *PAX6)* to establish whether any were associated with normal CCT variation in the general population. We hypothesized that common polymorphisms within these genes could contribute to normal variation in CCT, as opposed to the more deleterious mutations and knockouts seen in the human and animal models with extreme CCT values. To screen for the majority of known common genetic variation within these genes, we employed a tagging single nucleotide polymorphism (SNP) approach by selecting SNPs from the HapMap database. The cohort we used for this study was from a large, population-based study based in the Blue Mountains region, west of Sydney, Australia.

## Methods

### Participant recruitment

Participants were recruited as part of the Blue Mountains Eye Study (BMES), a population-based survey of vision and common eye diseases in the Blue Mountains region, west of Sydney, Australia. The participants recruited for this study were predominately Caucasian and the population has been described in detail previously [29]. As part of the ocular examination, CCT was measured using ultrasound pachymetry. Genomic DNA was extracted from peripheral blood according to standard methods. In total, there were 956 subjects with CCT measurements who were eligible to be included. The mean CCT of the BMES population was 539.7±32.8 µm and was normally distributed. Average age of the BMES cohort was 73.8 years, ranging from 60 to 95 years of age. Sex representation was slightly skewed with 59.9% of participants being female, while there were 62 (6.5%) confirmed cases of OAG within the cohort. Ethical approval was obtained from the relevant committees of the Westmead Millennium Institute at the University of Sydney (Sydney, Australia), as well as that from Flinders Medical Centre and Flinders University (Adelaide, Australia). The study adhered to the tenets of the Declaration of Helsinki.

### Genotyping of candidate genes in BMES cohort

For assessing the association of candidate genes with CCT, tagging SNPs were chosen from the Centre d’Etude du Polymorphisme Humain from Utah (CEU) population of the HapMap project [30], using Haploview v4.0 software [31]. Linkage disequilibrium blocks were defined using the ‘solid spine of LD’ function. An r^2^ threshold of 0.8 was set and SNPs with a minor allele frequency of <0.05 were excluded. The number of tagging SNPs selected for each gene was as follows, with the percentage of total genetic variation covered by the SNPs in parentheses; *AQP1*-10 (97.5)*; AQP5*-10 (97.6); *DCN*-10 (98.3); *FBN1*-8 (94.1); *KERA*-11 (94); *LUM-*11 (92.5); *OGN*-5 (98.4); and *PAX6*-7 (96.3).

Genotyping was performed using iPLEX GOLD chemistry (Sequenom Inc., San Diego, CA) on an Autoflex Mass Spectrometer (Sequenom Inc.) at the Australian Genome Research Facility, Brisbane, Australia. In the first phase of genotyping, tagging SNPs from all genes were genotyped in the extreme upper and lower quintiles (above the 80th or below the 20th percentile points) of the normal CCT distribution, drawn from the 956 subjects in the BMES. In the extreme upper quintile, all subjects had a CCT above 567.26 µm, while all subjects in the extreme lower quintile had a CCT below 510.75 µm. This methodology was used in the initial analysis to enrich for genetic effects contributing to the extremes of CCT variation, thus allowing us to prioritise SNPs to be screened in the full BMES cohort. Both the upper and lower quintiles contained 188 subjects each. Alleles and genotypes were tested for association with thin CCT using the software program PLINK v1.06 in a case-control design [32]. The second phase involved screening associated SNPs from the first phase in the remaining samples from the BMES cohort and analysis of the entire cohort using the quantitative trait CCT as the phenotype. An adjusted p value was calculated for each SNP using linear regression by incorporating both sex and age as covariates. Correction of p values for multiple testing was undertaken using the online SNPSpD interface [33,34]. The standard Bonferroni method of multiple testing correction is overly conservative, particularly when tests are not completely independent, as is the case here when SNPs are in linkage disequilibrium with each other. The correction method offered on the SNPSpD interface accounts for linkage disequilibrium between SNPs and determines the total number of independent tests per gene. Haplotypes from both experimental phases were estimated and association tested using HaploStats v1.3.1 software (Mayo Clinic, Rochester, MN). Haplotypes were primarily constructed based on LD block structure, although various combinations of sequential SNPs were also tested for all genes. Correction for multiple testing was performed using a standard Bonferroni adjustment for the total number of observed haplotypes. Statistical significance was accepted as p<0.05.

Power calculations were conducted for the case-control analysis using the Genetic Power Calculator [35]. Calculations were performed with risk allele frequencies of 0.2, 0.3, and 0.4. Genotype relative risks for the heterozygous and homozygous genotypes were set at 1.5 and 2.25, respectively. Linkage disequilibrium between the marker and risk allele was set at D’ prime=1.0. Under these criteria, the cohort had a power of 78.4% at a risk allele frequency of 0.2, 82.9% at 0.3 and 81.6% at 0.4 when calculated for a dominant model.

## Results

### Case-control analysis

The p values for both the dominant and recessive inheritance models of all the tagging SNPs typed in the extreme quintiles of the BMES distribution are shown in Table 1. Of the 71 total SNPs that were genotyped, 12 had a very low minor allele frequency and did not yield any association results. There were 6 SNPs in total that had a p value below the nominal significance level of 0.05; rs17352842 and rs9806323 (*FBN1*); rs11105956 and rs12231819 (*KERA*); rs2761678 (*OGN*); rs3026398 (*PAX6*). However, only one of these SNPs, rs17352842, survived correction for multiple testing. Using the modified correction value calculated by SNPSpD for *FBN1*, the adjusted p value for rs17352842 was determined to be 0.037. To decide whether to screen these six SNPs in the full BMES cohort, we also assessed whether there were any significant haplotype associations in the relevant genes. Both *FBN1* and *PAX6* also showed significant haplotype associations (data not shown), while no significant haplotypes involving *KERA* and *OGN* were observed. Thus, all SNPs from *FBN1* and *PAX6* were typed in the full cohort.

**Table 1:** Results of association tests for SNPs from all the candidate genes in the case-control analysis.

** **	** **	** **	**p value**		** **	** **	** **	**p value**	
**Gene**	**SNP**	**MAF**	**Dominant**	**Recessive**	**Gene**	**SNP**	**MAF**	**Dominant**	**Recessive**
*AQ1*	rs7788618	0.16	0.49	0.42	*KERA*	rs10745549	0.1	0.23	0.1
** **	rs1476597	0.44	0.82	0.6	** **	rs2701166	0.26	0.7	0.08
** **	rs1004317	0.36	1	0.5	** **	rs1920773	0.08	0.87	0.24
** **	rs17159702	0.24	0.059	0.085	** **	rs11105956	0.06	0.41	**0.041**
** **	rs1049305	0.39	0.85	0.62	** **	rs11105957	0.18	0.23	0.37
** **	rs13222180	0.24	0.18	0.57	** **	rs17018627	0.06	0.98	0.24
** **	rs10244884	0.48	0.16	0.89	** **	rs10859103	0.2	0.67	0.67
*AQ5*	rs3741559	0.2	0.59	0.93	** **	rs10859104	0.09	0.67	0.092
** **	rs461872	0.47	0.98	0.35	** **	rs12230223	0.17	0.48	0.53
** **	rs10875989	0.3	0.88	0.42	** **	rs12231819	0.09	0.51	**0.041**
** **	rs2878771	0.2	0.43	0.8	*LUM*	rs10745551	0.31	0.55	0.93
** **	rs3759129	0.22	0.77	0.8	** **	rs10777286	0.43	0.2	0.42
** **	rs296759	0.36	0.88	0.52	** **	rs10777287	0.11	0.19	0.24
** **	rs296763	0.23	0.15	0.16	** **	rs10859105	0.25	0.95	0.56
** **	rs1996315	0.43	0.35	0.8	** **	rs11105987	0.12	0.7	0.1
** **	rs2849266	0.08	0.19	0.1	** **	rs7976738	0.13	0.29	0.55
** **	rs10875990	0.06	0.096	0.24	** **	rs11478	0.08	0.53	0.96
*DCN*	rs12819853	0.3	0.67	0.85	** **	rs2268578	0.11	0.74	0.24
** **	rs17658598	0.15	0.77	0.32	*OGN*	rs7047089	0.33	0.11	0.85
** **	rs17658685	0.04	0.3	0.23	** **	rs10761156	0.49	0.46	0.071
** **	rs566806	0.25	0.91	1	** **	rs2761678	0.1	0.091	**0.018**
** **	rs741212	0.12	0.76	0.42	** **	rs2761680	0.12	0.4	0.54
** **	rs10492230	0.16	0.98	0.5	*PAX6*	rs3026398	0.28	**0.011**	0.15
*FBN1*	rs17352842	0.2	**0.0056**	0.45	** **	rs662702	0.05	0.3	0.24
** **	rs10519177	0.25	0.95	0.75	** **	rs2071754	0.21	0.18	0.3
** **	rs9806323	0.14	**0.033**	0.37	** **	rs3026390	0.49	0.24	0.18
** **	rs683282	0.32	0.51	0.75	** **	rs7942007	0.2	0.86	0.16
** **	rs12915677	0.17	0.57	0.36	** **	rs12286701	0.03	0.28	0.24
** **	rs17364665	0.05	0.2	0.24	** **	** **	** **	** **	** **
** **	rs591519	0.09	0.085	0.98	** **	** **	** **	** **	** **
** **	rs11854144	0.12	0.11	0.14	** **	** **	** **	** **	** **

Results of association tests for tagging SNPs from all candidate genes screened in the extreme lower and upper CCT quintiles derived from the 956 normal individuals of the entire BMES cohort. Both the lower and upper quintiles contained 188 subjects each. P values are given for both the dominant and recessive models, with values in bold considered significant at the p<0.05 level. In the table, MAF=minor allele frequency.

### Quantitative trait analysis

In total, 15 SNPs were genotyped in the full cohort, comprising eight from *FBN1* and seven from *PAX6.* Of these 15 SNPs, only two, rs17352842 from *FBN1* and rs3026398 from *PAX6*, showed any significant association with CCT in the full cohort. The genotype frequencies, mean CCT values for each genotype and associated p values for these two SNPs and the other significant *FBN1* SNP from the first phase genotyping (rs9806323) are illustrated in Table 2. As there were no significant associations among the remaining 13 SNPs, these are not shown, but the genotyping data from these SNPs was used to test for haplotype associations. The *FBN1* SNP rs17352842 was nominally associated with CCT under a dominant model, with an unadjusted p value of 0.02, with no change after adjustment for age and sex. This p value did not survive correction for multiple testing. The findings were similar for the *PAX6* SNP rs3026398, with a nominally significant p value of 0.02 under a dominant model in both the unadjusted and adjusted analysis. Again, this p value did not survive correction for multiple testing.

**Table 2:** Association of *FBN1* and *PAX6* SNPs with CCT in the full cohort.

** **	** **	** **	** **	** **	**Unadjusted p value**		**Adjusted p value**	
**Gene**	**SNP**	**Genotype**	**Frequency**	**Mean CCT±SD (µm)**	**Dominant**	**Recessive**	**Dominant**	**Recessive**
*FBN1*	rs17352842	C/C	0.63	541.3±32.4	**0.02**	0.81	**0.02**	0.94
** **	** **	C/T	0.32	535.9±32.4	** **	** **	** **	** **
** **	** **	T/T	0.05	538.2±32	** **	** **	** **	** **
** **	rs9806323	T/T	0.70	541.6±33.2	0.09	0.68	0.11	0.59
** **	** **	T/A	0.27	536.8±31.8	** **	** **	** **	** **
** **	** **	A/A	0.03	543±29.3	** **	** **	** **	** **
*PAX6*	rs3026398	G/G	0.50	536.7±33.3	**0.02**	0.8	**0.02**	0.72
** **	** **	G/A	0.42	542.1±31.9	** **	** **	** **	** **
** **	** **	A/A	0.08	540.2±30.7	** **	** **	** **	** **

Genotype frequencies and mean CCT values for each genotype of selected SNPs from *FBN1* and *PAX6* typed in 956 subjects from the full BMES cohort. P values are calculated for both dominant and recessive modes of inheritance with the adjusted p value corrected for sex and age. Values in bold are considered significant at the p<0.05 level. In the table, SD=standard deviation.

Results of the haplotype analysis performed on *FBN1* and *PAX6* can be seen in Table 3 and Table 4. The CT haplotype in *FBN1*, which comprises SNPs rs17352842 and rs9806323, was significantly associated with CCT under an additive and recessive model. However, following Bonferroni correction for the four observed haplotypes, only the value under the recessive model remained significant at p=0.048. A two SNP haplotype in *PAX6* comprising rs3026398 and rs662702 was found to be significantly associated with CCT under all three inheritance models. The strongest association was seen with the GC haplotype under a recessive model, which gave a p value of 0.009 following correction for the three observed haplotypes. Both the GC haplotype under an additive model and the AC haplotype under a dominant also yielded p values that survived Bonferroni correction for multiple testing.

**Table 3:** Association of haplotypes in *FBN1* with CCT in the full cohort.

** **	**SNP**		** **	**p value**		
**Haplotype**	**1**	**2**	**Frequency**	**Additive**	**Dominant**	**Recessive**
1	T	A	0.10	0.138	0.073	0.534
2	T	T	0.11	0.147	0.133	0.810
3	C	A	0.06	0.548	0.580	NA
4	C	T	0.73	0.027	0.589	**0.012**

A two SNP haplotype was constructed from *FBN1* SNPs using rs17352842 (SNP 1) and rs9806323 (SNP 2). This haplotype was assessed in 956 subjects from the full BMES cohort and tested for association with CCT as a continuous variable. Additive, dominant and recessive modes of inheritance were analyzed and values in bold survived Bonferroni correction for the four observed haplotypes. Values were considered significant at the p<0.05 level following correction.

**Table 4:** Association of haplotypes in *PAX6* with CCT in the full cohort.

** **	**SNP**		** **	**p value**		
**Haplotype**	**1**	**2**	**Frequency**	**Additive**	**Dominant**	**Recessive**
1	G	C	0.63	**0.008**	0.369	**0.003**
2	G	T	0.06	0.301	0.326	0.678
3	A	C	0.31	0.025	**0.009**	0.579

A two SNP haplotype was constructed from *PAX6* SNPs using rs3026398 (SNP 1) and rs662702 (SNP 2). This haplotype was assessed in 956 subjects from the full BMES cohort and tested for association with CCT as a continuous variable. Additive, dominant and recessive modes of inheritance were analyzed and values in bold survived Bonferroni correction for the three observed haplotypes. Values were considered significant at the p<0.05 level following correction.

## Discussion

The genetic factors involved in the determination of CCT are largely unknown. This is despite several familial and twin studies indicating that CCT exhibits a very strong hereditary component [9-11]. These characteristics make CCT ideal for a candidate gene study, particularly given that as with any quantitative trait, CCT is likely to be under the regulation of numerous genes. For this study, we selected eight different candidate genes that we hypothesized to be associated with CCT. The selected genes can be divided into three broad groups based on their similarities; the aquaporins - *AQP1* and *AQP5*; SLRPs of the cornea - *DCN*, *KERA*, *LUM*, *OGN*; and genes associated with abnormal CCT in disease states - *FBN1* and *PAX6*. Our hypothesis was that non-pathogenic polymorphisms within these genes could be associated with the normal CCT variation seen within the general population and our approach to find these variants was to select a range of tagging SNPs that would cover the majority of genetic variation within these genes.

Both *AQP1* and *AQP5* are water channel proteins expressed in the mammalian cornea, including human cornea [36-38]. *AQP1* is expressed in the corneal endothelium while *AQP5* is found in the epithelium and they both play an important role in the regulation of fluid transport, which is essential for maintaining corneal transparency. To further characterize the function of these aquaporins, Thiagarajah et al. [28] studied the corneas of mice with *Aqp1* and *Aqp5* gene knockouts. They observed that the *Aqp1* null mice had a significantly reduced corneal thickness, while the *Aqp5* mice had a significantly increased corneal thickness. The mechanism responsible for this change is likely to be alterations in the water balance of the corneal stroma, with dehydration causing thinning of the cornea and swelling resulting in a thickening of the cornea. These observations were the basis for our inclusion of *AQP1* and *AQP5* in our candidate gene study, as we hypothesized that minor alterations in the water balance of the cornea caused by polymorphisms in *AQP1* and *AQP5* may account for some of the normal variation in CCT in humans. However, our genotyping results indicated that there was no association with these genes and CCT variation.

This study also examined several genes that code for SLRPs, a class of proteins that are vital to the development of a functional cornea. SLRPs are macromolecules composed of a protein core with covalently linked glycosaminoglycan side chains, and constitute a major component of the extracellular matrix, where they form complexes with other matrix proteins such as collagen [39]. The corneal stroma, which accounts for approximately 90% of the total thickness of the cornea, is predominately an extracellular tissue, comprised of a highly ordered and uniform array of collagen fibrils, features which are essential for establishing corneal transparency. Corneal SLRPs play a crucial role in assembling this array. More specifically, *DCN*, *KERA*, *LUM*, and *OGN* are all involved in regulating the diameter and growth of these collagen fibrils, as well as establishing uniform inter-fibrillar spacing [40]. Alterations in the diameter of collagen fibrils could be of importance in a human model, where a narrower collagen fibril diameter has been shown to directly correlate with a reduction in CCT [41,42]. As discussed previously, there is also evidence from both human and mouse studies indicating that mutations or knockouts of these SLRP genes directly influence CCT [23-26]. This data provided the foundation of our hypothesis that the aforementioned SLRPs could be associated with normal CCT variation. The results of our genotype screening however, suggests that these genes are not involved in CCT determination, with no SNPs surviving multiple testing correction and no significantly associated haplotypes.

Two genes were also selected as candidates in our study based on their causal involvement with diseases that present with abnormal CCT measurements. *FBN1* is an extracellular glycoprotein that is a constituent of the 8–10 nm microfibrils that provide structural support to basement membranes and elastic and non-elastic tissues [43]. Expression of *FBN1* is widespread throughout the body and has been documented in the cornea [44,45]. Mutations in *FBN1* can cause Marfan syndrome, a connective tissue disorder that results in a range of clinical manifestations, with the principal complications occurring in the eye and the skeletal and cardiovascular systems [19,20]. Studies have shown that Marfan syndrome patients also have a lower CCT [15,16]. The other candidate gene that was selected based on its association with abnormal CCT measurements in disease was *PAX6*, a transcription factor that plays an integral role in the early development of the ocular, central nervous and endocrine systems [46,47]. Mutations in *PAX6* have been shown to result in aniridia, a congenital ocular condition which presents with abnormalities including iris hypoplasia, corneal opacities, cataracts, and foveal and optic nerve hypoplasia [21,22]. Aniridia has also been associated with a significantly thicker CCT [17,18]. It has been postulated that mutations in *PAX6* may disrupt the normal homeostasis of corneal stromal keratocytes, leading to increased stromal extracellular matrix synthesis and subsequent increases in CCT [18]. While pathogenic mutations within *FBN1* and *PAX6* can be directly correlated with alterations in CCT, these variants would not contribute to normal CCT variation. Therefore, we hypothesized that non-pathogenic polymorphisms in either of these genes could be involved in the more subtle variation in CCT seen in the general population.

Results from the first phase of genotyping indicated that SNPs from both *FBN1* and *PAX6* were suitable for screening in the full BMES cohort. While only rs17352842 from *FBN1* survived correction for multiple testing, there were some significant haplotype associations that ensured both genes warranted further investigation. Analysis of all the *FBN1* and *PAX6* SNPs in the full BMES cohort however, did not strengthen the argument that these genes are associated with CCT variation. The p values from all the significant SNPs in the first phase genotyping, rs17352842 and rs9806323 from *FBN1* and rs3026398 from *PAX6*, weakened when assessed in the larger cohort. The reduction in significance is an interesting observation and could be the result of several factors. The increased power provided by genotyping the SNPs in an additional 580 samples suggests that the initial analysis was under-powered and that the associations seen were possibly type I error. Alternatively, the first phase of genotyping in the upper and lower quintiles could have enriched for variants that are only important in the extreme ends of the CCT spectrum and thus, by introducing the samples from the ‘middle’ of the distribution, potentially real associations could be weakened. This is a potential weakness of the approach we undertook to prioritize SNPs from the first phase screen, as we may have ignored SNPs that could have been significantly associated had they been screened in the full BMES cohort. However, the reduced cost of genotyping the extremes in the first instance enabled us to screen more candidate genes.

The haplotype analysis conducted on the full cohort provided additional evidence for an association of these genes with CCT, with haplotypes from both *FBN1* and *PAX6* yielding significant p values that survived Bonferroni correction. The results suggest there may be variants within these regions of *FBN1* and *PAX6* that are involved in CCT determination, particularly given that the haplotypes for both genes contained the most significantly associated SNPs. Given the relatively strong haplotype data, it is intriguing that single SNP analysis showed a weak or no association. However, studies have demonstrated that examining haplotypes can be superior to single SNP analysis when undertaking a candidate gene study and it is possible that the haplotypes are detecting variation within these genes that cannot be ascertained from investigating single SNPs [48,49]. This scenario also presents an inherent difficulty with the haplotype and tagging SNP methodologies, whereby the actual causative variants remain unknown. The contradicting data presented by the single SNP and haplotype analyses of *FBN1* and *PAX6* ensures that further work is needed to assess the potential involvement of these genes with CCT determination. Replication of these results in other cohorts is required to confirm the findings of this study.

The identification of CCT as a major risk factor for OAG gives major clinical relevance to understanding the genetic mechanisms of this trait. OAG is an adult-onset, irreversible cause of vision loss that will affect an estimated 58 million people worldwide by 2020, making it the second leading cause of blindness after cataract [50]. Due to its increasing prevalence with age, the burden of OAG on society is likely to increase substantially as our population ages. In Australia alone, the estimated annual cost to the economy from OAG will be $4.3 billion by 2025 [51]. Therefore, improving current methods of diagnosis and treatment for this condition remains a priority. Vital to achieving this aim is the enhancement of our understanding of the genes that predispose to OAG. Despite evidence from familial and ethnic studies demonstrating the strong genetic component underlying OAG, this facet of the disease remains poorly characterized [52]. Only one major gene, myocilin (*MYOC*), has been identified to-date and mutations in this gene only account for approximately 3% of cases [53]. With substantial evidence supporting low CCT as a risk factor for OAG, it is conceivable that genes may be common to both pathways. A biological link may exist between aspects of the cornea that regulate its thickness and the physical and structural properties of tissues involved in glaucoma pathogenesis, such as the lamina cribrosa or trabecular meshwork. Even if no such biological interaction exists and low CCT is a risk factor purely due to tonometry artifact, developing our knowledge of the genetic mechanisms involved in CCT variation will improve our understanding of the nature of the association between CCT and OAG.
